# Association Between Serum Vitamin D and Albuminuria in Type 2 Diabetes Independent of Inflammatory Markers and Renal Function

**DOI:** 10.1002/edm2.70093

**Published:** 2025-08-13

**Authors:** Parisa Farshchi, Sahar Karimpour Reyhan, Mahsa Abbaszadeh, Soghra Rabizadeh, Alireza Esteghamati, Nasim Khajavi Rad, Soheil Karimpour Reyhan, Elahe Saffari, Manouchehr Nakhjavani

**Affiliations:** ^1^ Department of Internal Medicine, Imam Khomeini Hospital Complex, School of Medicine Tehran University of Medical Sciences (TUMS) Tehran Iran; ^2^ Endocrinology and Metabolism Research Center (EMRC), Vali‐Asr Hospital Tehran University of Medical Sciences (TUMS) Tehran Iran; ^3^ Mechanical Engineering School University of Tehran Tehran Iran; ^4^ Isfahan University of Medical Sciences Isfahan Iran

**Keywords:** albuminuria, diabetic nephropathy, hs‐CRP, T2D, TNF‐α25(OH) vitamin D

## Abstract

**Introduction:**

To explore the relationship between serum high‐sensitivity C‐reactive protein (hs‐CRP), tissue necrosis factor‐α (TNF‐α) and 25‐Hydroxyvitamin D (25(OH) vitamin D) with albuminuria in patients with type 2 diabetes mellitus (T2D).

**Methods:**

This was a cross‐sectional study of 86 T2D patients divided into categories of with and without albuminuria based on the urine albumin‐to‐creatinine ratio (UACR). A 25(OH) vitamin D concentration ≤ 15 ng/mL was defined as vitamin D deficiency, within 15–30 ng/mL as vitamin D insufficiency, and > 30 ng/mL as serum 25(OH) vitamin D sufficiency. A hs‐CRP level ≤ 2.5 mg/L was considered low, whereas a hs‐CRP level > 2.5 mg/L was considered high. TNF‐α was classified as low or high with an 8.2 pg/mL cutoff level based on receiver operating characteristic (ROC) curve analysis. P values < 0.05 were considered to be significantly associated with albuminuria.

**Results:**

Vitamin D deficiency was significantly more commonly observed among T2D patients with albuminuria than those without albuminuria (adjusted OR = 7.34, 95% CI = 2.3–23.6, *p* = 0.001). Higher serum TNF‐α levels (TNF‐α > 8.2 pg/mL) were more frequently associated with the presence of albuminuria in T2D patients (adjusted OR = 6.77, 95% CI = 1.61–28.4; *p =* 0.009). Similarly, elevated serum hs‐CRP levels (hs‐CRP > 2.5 mg/L) were more commonly found among patients with T2D and albuminuria than in those without (adjusted OR = 4.7, 95% CI = 1.4–15.8; *p =* 0.012).

**Conclusions:**

Vitamin D deficiency is a significant correlate of albuminuria in T2D patients, independent of glomerular filtration rate (GFR) and basic inflammatory markers including hs‐CRP and TNF‐α. Moreover, serum hs‐CRP > 2.5 mg/L and TNF‐α > 8.2 pg/mL were each individually associated with a significantly increased likelihood of albuminuria in T2D patients.

## Graphical abstract

Vitamin D deficiency is a significant correlate of albuminuria in T2D patients, independent of eGFR and basic inflammatory markers including hs‐CRP and TNF‐α. Serum hs‐CRP > 2.5 mg/L and TNF‐α > 8.2 pg/mL were each individually associated with a significantly increased likelihood of albuminuria in T2D patients.

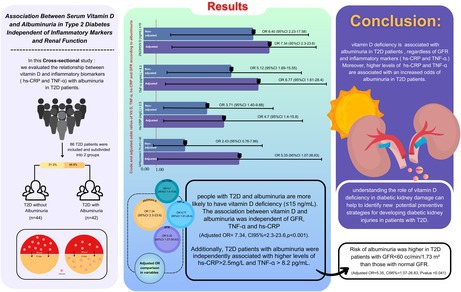

## 1 Introduction

Vitamin D is a fat‐soluble steroid hormone with a wide spectrum of functions extending beyond calcium‐phosphate homeostasis and bone metabolism [1]. These pivotal activities include the regulation of renal function and modulation of immune and inflammatory responses that are mediated through vitamin D–vitamin D receptor (VDR) interactions [2, 3, 4].

Vitamin D has renal protective effects through two major mechanisms—the inhibition of the renin‐angiotensin system (RAS) and the nuclear factor kappa B (NF‐κB) route—both of which act as salient keys in renal diseases by enhancing inflammation and fibrogenesis [2]. Vitamin D represses renin gene transcription by disrupting the cyclic adenosine monophosphate (cAMP) signalling trajectory; thus, it alters renin and angiotensin II biosynthesis, which are responsible for vasoconstriction, oxidative stress, inflammation, fibrogenesis, and subsequent renal injury [5]. Studies have demonstrated the role of vitamin D in the suppression of the NF‐κB pathway through disruption of NF‐κB DNA binding. Consequently, the NF‐κB pathway cannot regulate the immune response or inflammatory cytokines that are involved in the development of kidney diseases [6].

Recent investigations have reported potential negative correlations between serum 25‐Hydroxyvitamin D (25(OH) vitamin D) and various proinflammatory cytokines as well as the risk of type 2 diabetes mellitus (T2D) [7, 8]. On the basis of a systematic review carried out by Authier et al., in general, prospective studies have shown moderate to strong decreases in the serum markers of inflammation and glucose metabolism disorders associated with augmented 25(OH) vitamin D levels. Nonetheless, interventional studies have shown that vitamin D supplement consumption has no or negligible impact on these disorders [1]. Furthermore, in some studies, the serum vitamin D concentration was negatively related to augmented renal inflammation in patients with different kidney problems [9, 10, 11, 12].

High blood glucose induces several pathological pathways in the kidney, which include the previously mentioned RAS and NF‐κB pathways that cause renal damage [2]. Considering the inhibitory role of vitamin D on the RAS and NF‐κB pathways, it has been illustrated that a low level of serum 25(OH) vitamin D may affect glucose and insulin metabolism [13]. With respect to glucose homeostasis, vitamin D influences pancreatic beta‐cell propagation and longevity and the capacity to react to increased insulin demand, for instance, in patients with T2D [14]. Vitamin D deficiency might diminish insulin secretion and increase peripheral insulin resistance and blood glucose levels [13, 15]. However, angiotensin II activates NF‐κB, while NF‐κB mediates high glucose‐induced angiotensinogen expression in renal cells. This domino effect exacerbates the local inflammation of angiotensin II in diabetic nephropathy [6].

Recently, the role of inflammation in initiating diabetic nephropathy has been highlighted [16, 17, 18]. In a study performed by Preciado‐Puga et al., serum tissue necrosis factor‐α (TNF‐α) was suggested to be a potential factor for the progression of T2D complications [19]. Similarly, Moriwaki et al. showed increased levels of TNF‐α and interleukin −18 (IL‐18) in diabetic patients with albuminuria [20]. Vandana Varma also reported elevated serum C‐reactive protein (CRP) levels in patients with diabetic nephropathy [21]. However, investigations by Lampropoulou and Cao L et al. in different diabetic populations did not support these previous results; they did not observe any marked difference in the serum TNF‐α concentration between patients with diabetic nephropathy and patients without diabetic nephropathy [22, 23].

Despite recent research in the field of diabetic nephropathy and research on the role of vitamin D and inflammatory biomarkers in the pathogenesis of diabetic nephropathy, there are still many unknowns in this area, with some controversial and inconclusive results. Moreover, the potential effect and interaction of vitamin D and inflammatory biomarkers on the initiation and progression of diabetic nephropathy are still unclear. Thus, further studies are needed to provide more convincing results. In this research, we investigated the role of vitamin D and basic inflammatory biomarkers, serum highly sensitive CRP (hs‐CRP) and TNF‐α, and their possible interactions in diabetic kidney disease in an Iranian population.

## 2 Materials and Methods

### 2.1 Study Design and Population

In this cross‐sectional study, 86 patients with T2D, diagnosed based on the American Diabetes Association criteria [24] and referred to the Diabetes Clinic of Tehran University of Medical Sciences, were recruited. Eligible participants were adults aged 30 years or older with a confirmed diagnosis of T2D for at least one year. To ensure internal validity and reduce potential sources of bias, we applied comprehensive exclusion criteria. Patients were excluded if they had type 1 diabetes mellitus, renal disease unrelated to diabetic nephropathy, malignancy, advanced liver disease, pregnancy or lactation, or a diagnosis of any acute or chronic infections, autoimmune, or rheumatologic conditions. Additionally, individuals who had been hospitalised in the past three months, or who had received vitamin D supplementation exceeding 2000 IU/day, corticosteroids, NSAIDs, or immunosuppressive therapy within the past three months, were excluded. These measures were taken to avoid confounding by factors that might independently influence inflammation or vitamin D metabolism.

We systematically collected detailed clinical and demographic data through structured interviews and review of medical records. This included age, sex, duration of diabetes, and detailed information on comorbid conditions and current medication use. To control for potential confounding factors, all relevant variables–such as comorbidities and medication use–were included in multivariate regression models. Sensitivity analyses were also conducted by excluding participants with major comorbidities to test the robustness of the observed associations. These analytical strategies allowed for a more accurate evaluation of the independent associations between vitamin D status, inflammatory markers, and albuminuria, minimising the potential influence of confounding variables.

Participants were categorised into two groups. Forty‐two subjects who had a urine albumin‐to‐creatinine ratio (UACR) ≥ 30 mg/g were assigned to the T2D with albuminuria group, and 44 subjects who had a UACR < 30 mg/g were considered as the T2D without albuminuria group.

### 2.2 Measurements

Demographic information such as age and sex, as well as anthropometric data including height (cm) and weight in light clothing (kg), was collected. Body mass index (BMI) [kg/m^2^] was computed by dividing weight by height squared using the Quetelet formula. Moreover, blood pressure (BP) was measured twice after 10 min of rest and 5 min apart. For each subject, the average of the two recorded blood pressure measurements was considered the patient's BP.

Blood samples were collected after 12 h of overnight fasting. Fasting blood sugar (FBS) [mg/dL], 2‐h postprandial glucose (2HPPG) [mg/dL], creatinine [mg/dL], low‐density lipoprotein cholesterol [LDL‐C] [mg/dL], high‐density lipoprotein cholesterol [HDL‐C] [mg/dL], cholesterol [mg/dL] and triglyceride (TG) [mg/dL] were analysed via the enzymatic method (GOD‐POD) Delta Darman Part Lab Test, Iran; glycosylated haemoglobin (HbA_1_c) [%] was tested via direct turbidimetry (Archem Diagnostics, Turkey); 25(OH) vitamin D [ng/mL] was analysed via an ELISA method via a Euroimmun analyser (Euroimmun, Germany); tissue necrosis factor alpha (TNF‐α) [pg/mL] was measured to determine the ELISA level using a Diaclone Analyse (diaclone, France); and high‐sensitivity C‐reactive protein (hs‐CRP) [mg/L] was analysed via an ELISA method via a Monobind Analyser (Monobind Analyser, Monobind, USA). Additionally, two random morning urine samples were obtained from each patient to measure urine albumin (mg/dL) and urine creatinine (gr/dL) levels via the colorimetric method (Jaffe) in accordance with the ZiestChem Diagnostics, Iran Kit; these levels were subsequently used to calculate the urine albumin‐to‐creatinine ratio (UACR) [mg/g].

The estimated glomerular filtration rate (eGFR) [cc/min/1.73m^2^] was calculated for each participant using the 2021 CKD‐EPI creatinine‐based equation as follows: [142 × min(Scr / 0.7, 1)^(−0.241)^ × max(Scr / 0.7, 1)^(−1.200)^ × 0.9938^Age^ × 1.012] for females; and [142 × min(Scr / 0.9, 1)^(−0.302)^ × max(Scr / 0.9, 1)^(−1.200)^ × 0.9938^Age^] for males, where Scr is serum creatinine [mg/dL], and age is expressed in years.

### 2.3 Ethical Considerations

This research was endorsed by Tehran University of Medical Sciences with the ethical code IR.TUMS.IKHC.REC.1398.180. Written authorization was obtained from all study participants. This study was conducted in accordance with the Declaration of Helsinki.

### 2.4 Statistical Analysis

To facilitate clinical interpretation and minimise the influence of outliers, continuous variables were categorised based on clinical guidelines or data‐driven thresholds. Serum 25(OH) vitamin D levels were classified according to Kidney Disease Outcomes Quality Initiative (K/DOQI) guidelines [25] into deficiency (≤ 15 ng/mL), insufficiency (15–30 ng/mL), and sufficiency (> 30 ng/mL). For analytical purposes, and due to sample size limitations, and in line with previous studies in diabetic populations, we combined the insufficiency and sufficiency categories, as they are both considered to be above the threshold of deficiency with respect to clinical bone and mineral metabolism outcomes.

A cutoff of 2.5 mg/L was used to dichotomize hs‐CRP levels based on the work of Pepys and Hirschfield [26], widely referenced in the context of low‐grade inflammation and cardiometabolic risk. As there is no universally accepted clinical cutoff, an optimal threshold of 8.2 pg/mL was derived via receiver operating characteristic (ROC) curve analysis to best differentiate patients with versus without albuminuria in our sample. To determine the optimal cutoff for serum TNF‐α in predicting albuminuria, we conducted a ROC curve analysis using UACR ≥ 30 mg/g as the binary classification outcome. The Youden index was applied to identify the TNF‐α level that maximised the sum of sensitivity and specificity. ROC curve analysis for serum TNF‐α yielded an area under the curve (AUC) of 0.72, suggesting a fair ability to distinguish between patients with and without albuminuria. The optimal cutoff point identified by the Youden index was 8.2 pg/mL, with a sensitivity of 71.4% and specificity of 68.2%. eGFR values were categorised using a 60 cc/min/1.73 m^2^ cutoff to stratify participants according to established thresholds for chronic kidney disease (CKD), as recommended by current clinical guidelines. This classification facilitates the identification of individuals with clinically significant reductions in renal function, irrespective of albuminuria status, thereby enhancing the diagnostic accuracy and clinical applicability of the study outcomes.

All the statistical analyses were carried out with SPSS software, version 25. After presenting the variables as proportions with 95% confidence intervals (CIs), we conducted bivariate logistic regression analyses (Tables 1 and 2) to assess the association between clinical and biochemical parameters and the presence of albuminuria in patients with T2D. The variables analysed included FBS, 2HPPG, HbA1c, lipid profile components, BMI, systolic and diastolic blood pressure, gender, age, duration of diabetes, eGFR, TNF‐α, hs‐CRP, and serum vitamin D levels. Variables with a *p* value ≤ 0.20 in the bivariate analysis—specifically 2HPPG, eGFR, hs‐CRP, vitamin D, and TNF‐α—were considered potential candidates for multivariable logistic regression modelling (Table 3). This inclusive threshold was chosen to reduce the risk of excluding potentially important variables that may not show significance in unadjusted analysis but could have independent effects in a multivariable context. A backward stepwise elimination approach was used in the multivariable model, with variables retained if they remained statistically significant at *p* < 0.05. This modelling strategy aimed to identify independent predictors of albuminuria while minimising overfitting and ensuring model parsimony. Moreover, we used a Pearson correlation coefficient model to analyse the associations of serum TNF‐α and hs‐CRP with other variables in patients with and without albuminuria.

**TABLE 1:** Demographic and clinical characteristics of patients with T2D (bivariate analysis).

Variables	T2D without albuminuria	T2D with albuminuria	OR (95% CI)^b^	*p* ^b^
	*N* (%)^a^	*N* (%)^a^		
Patients	44 (51.2)	42 (48.8)		
Age group				0.60
34–55 years	19 (43.2)	20 (47.6)	—	
56–65 years	15 (34)	16 (38.1)	—	
> 65 years	10 (22.8)	6 (14.3)	0.99 (0.38–2.5)	
Gender				0.67
Male	24 (54.5)	21 (50)	—	
Female	20 (45.5)	21 (50)	0.83 (0.36–1.95)	
Duration of diabetes				0.90
≤ 15 years	33 (75)	31 (73.8)	—	
> 15 years	11 (25)	11 (26.2)	0.93 (0.36–2.48)	
BMI				0.77
< 25 kg/m2	16 (36.3)	14 (33.3)	0.88 (0.36–2.13)	
≥ 25 kg/m2	28 (63.7)	28 (66.7)	—	
Systolic blood pressure				0.40
< 140 mmHg	33 (75)	28 (66.7)	—	
≥ 140 mmHg	11 (25)	14 (33.3)	0.67 (0.26–1.70)	
Diastolic blood pressure				0.95
< 90 mmHg	41 (93)	39 (92.9)	—	
≥ 90 mmHg	3 (7)	3 (7.1)	0.95 (0.18–5.0)	

Abbreviations: 95% CI, 95% confidence interval; BMI, body mass index; OR, odds ratio; T2D, type 2 diabetes mellitus.

^**a**^ *N* (%): Number (percentage) of participants.

^**b**^ Results of bivariate analysis.

**TABLE 2:** Results of biochemical laboratory tests in patients with T2D (bivariate analysis).

Variables	T2D without albuminuria	T2D with albuminuria	OR (95% CI)^b^	*p* ^b^
	*N* (%)^a^	*N* (%)^a^		
Patients	44 (51.2)	42 (48.8)		
FBS [mg/dL]				0.34
≤ 130	11 (25)	7 (16.7)	0.60 (0.21–1.73)	
> 130	33 (75)	35 (83.3)	—	
2HPPG [mg/dL]				0.11
≤ 200	20 (45.5)	12 (28.6)	0.48 (0.20–1.17)	
> 200	24 (54.5)	30 (71.4)	—	
HbA_1_C [%]				0.91
≤ 7	10 (22.7)	10 (23.8)	1.06 (0.39–2.89)	
> 7	34 (77.3)	32 (76.2)	—	
LDL‐C [mg/dL]				0.99
≤ 100	23 (52.3)	22 (52.4)	1.00 (0.43–2.34)	
> 100	21 (47.7)	20 (47.6)	—	
HDL‐C [mg/dL]				0.70
≤ 35	6 (13.6)	7 (16.7)	—	
> 35	38 (86.4)	35 (83.3)	1.27 (0.39–4.14)	
TG [mg/dL]				0.79
≤ 150	26 (59)	26 (61.9)	1.13 (0.47–2.68)	
> 150	18 (41)	16 (38.1)	—	
GFR [cc/min/1.73m^2^]				0.14
< 60	5 (11.4)	10 (23.8)	2.43 (0.76–7.86)	
≥ 60	39 (88.6)	32 (76.2)	—	
25 (OH)Vit D [ng/mL]				< 0.001
≤ 15	7 (16)	23 (54.8)	6.40 (2.23–17.58)	
> 15	37 (84)	19 (45.2)	—	
TNF‐α [pg/mL]				0.002
≤ 8.2	18 (41)	5 (11.9)	—	
> 8.2	26 (59)	37 (88.1)	5.12 (1.69–15.55)	
Hs‐CRP [mg/L]				0.008
≤ 2.5	38 (86.4)	24 (57.1)	—	
> 2.5	6 (13.6)	18 (42.9)	3.71 (1.40–9.88)	

Abbreviations: 25 (OH) vitamin D, 25‐hydroxy vitamin D; 2HPPG, 2‐hourh postprandial glucose; 95% CI, 95% confidence interval; FBS, fasting blood glucose; GFR, glomerular filtration rate; HbA_1_C, glycosylated haemoglobinhemoglobin; HDL‐C, high‐density lipoprotein cholesterol; hs‐CRP, high‐sensitivity C‐reactive protein; LDL‐C, low‐density lipoprotein cholesterol; OR, odds ratio; TG, triglyceride; TNF‐α, tissue necrosis factor alpha.

^**a**^ *N* (%): Number (percentage) of participants.

^**b**^ Results of bivariate analysis.

**TABLE 3:** Results of biochemical laboratory tests in patients with T2D (Multivariate Analysis).

Variables	T2D without albuminuria	T2D with albuminuria	Adjusted OR (95% CI)^b^	*p* ^b^
	*N* (%)^a^	*N* (%)^a^		
Patients	44 (51.2)	42 (48.8)		
2HPPG [mg/dL]				0.39
≤ 200	20 (45.5)	12 (28.6)	1.62 (0.54–4.88)	
> 200	24 (54.5)	30 (71.4)	—	
GFR [cc/min/1.73m^2^]				0.041
< 60	5 (11.4)	10 (23.8)	5.35 (1.07–26.83)	
≥ 60	39 (88.6)	32 (76.2)	—	
25(OH)Vit D [ng/mL]				0.001
≤ 15	7 (15.9)	23 (54.8)	7.34 (2.3–23.6)	
> 15	37 (84.1)	19 (45.2)	—	
TNF‐α [pg/mL]				0.009
≤ 8.2	18 (40/9)	5 (11.9)	—	
> 8.2	26 (59.1)	37 (88.1)	6.77 (1.61–28.4)	
Hs‐CRP [mg/L]				0.012
≤ 2.5	38 (86.4)	24 (57.1)	—	
> 2.5	6 (13.6)	18 (42.9)	4.7 (1.4–15.8)	

Abbreviations: 25 (OH) Vit D, 25‐hydroxy vitamin D; 2HPPG, 2‐Hour Postprandial Glucose; 95% CI, 95% confidence intervals; GFR, glomerular filtration rate; hs‐CRP, High‐sensitivity C reactive protein; OR, Odds Ratios; TNF‐α, tissue necrosis factor alpha.

^a^ *N* (%): Number (percentage) of participants.

^b^ Results of multivariate analysis.

## 3 Results

### 3.1 Demographic and Clinical Characteristics

The demographic and clinical characteristics of the study population are presented in Table 1. The mean age of the participants was 57.64 ± 10.48 years, with the youngest and oldest participants being 34 and 85 years old, respectively. The duration of T2D ranged from one to 30 years. There were no statistically significant differences in age, sex, duration of diabetes, BMI, or blood pressure between the groups with and without albuminuria (*p* value > 0.05).

### 3.2 Results of Biochemical Lab Tests

The laboratory test results of all participants are reported in Tables 2 and 3. Vitamin D deficiency was significantly more commonly observed among patients with T2D with albuminuria than among T2D patients without albuminuria (adjusted OR = 7.34, 95% CI = 2.3–23.6, *p* = 0.001). Higher serum TNF‐α levels (TNF‐α > 8.2 pg/mL) were more frequently associated with the presence of albuminuria in patients with T2D (adjusted OR = 6.77, 95% CI = 1.61–28.4; *p =* 0.009). Similarly, elevated serum hs‐CRP levels (hs‐CRP > 2.5 mg/L) were more commonly found among patients with T2D and albuminuria than in those without (adjusted OR = 4.7, 95% CI = 1.4–15.8; *p =* 0.012). In addition, lower eGFR (eGFR < 60 cc/min/1.73m^2^) was associated with greater odds of albuminuria (adjusted OR = 5.35, 95% CI = 1.07–26.83; *p* = 0.041). After comprehensive adjustment for potential confounders, the mentioned association between serum vitamin D levels, TNF‐α, hs‐CRP, eGFR, and albuminuria remained statistically significant. There were no significant differences in the serum FBS, 2HPPG, HbA_1_C, HDL‐C, LDL‐C, or TG levels between T2D patients with and without albuminuria (*p value* > 0.05).

### 3.3 Correlations Between Inflammatory Markers and Other Variables

**FIGURE 1:**
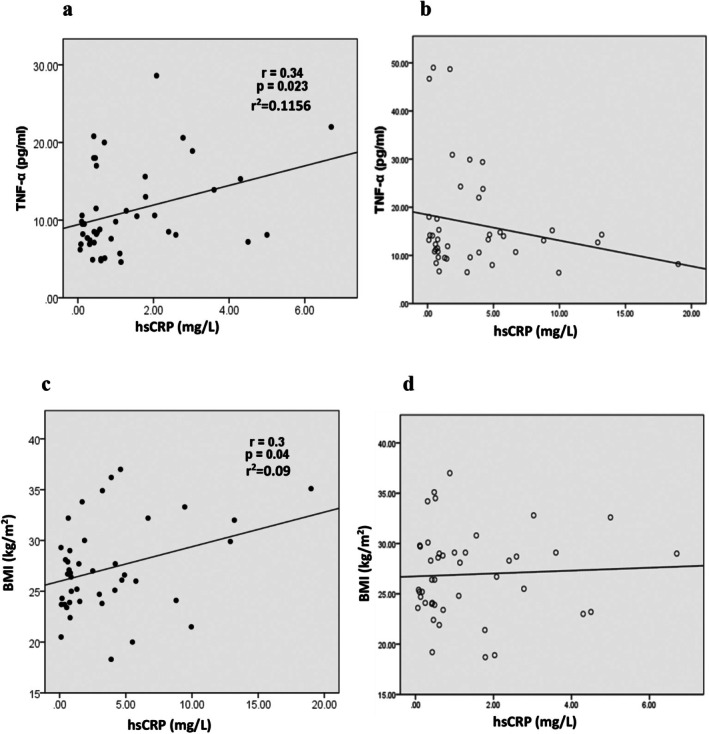
Correlation between (a) hs‐CRP and TNF‐α in patients with T2D without albuminuria, and between (b) hs‐CRP and TNF‐α in patients with T2D with albuminuria. Correlation between (c) hs‐CRP and BMI in patients with T2D with albuminuria, and between (d) hs‐CRP and BMI in patients with T2D without albuminuria.

There was a statistically significant but weak positive correlation between the serum TNF‐α concentration and the serum hs‐CRP concentration in patients with T2D without albuminuria (*r* = 0.34, *r* ^2^ = 0.1156; *p value* = 0.023) (Figure 1a). Additionally, there was a weak correlation between serum hs‐CRP and BMI in T2D patients with albuminuria (*r* = 0.3, *r* ^2^ = 0.09; *p value* = 0.04) (Figure 1c). However, we did not observe any relationship between inflammatory markers (TNF‐α or hs‐CRP) and other variables, including the serum 25(OH) vitamin D concentration, in patients with T2D (Figure 1b,d).

**FIGURE 2:**
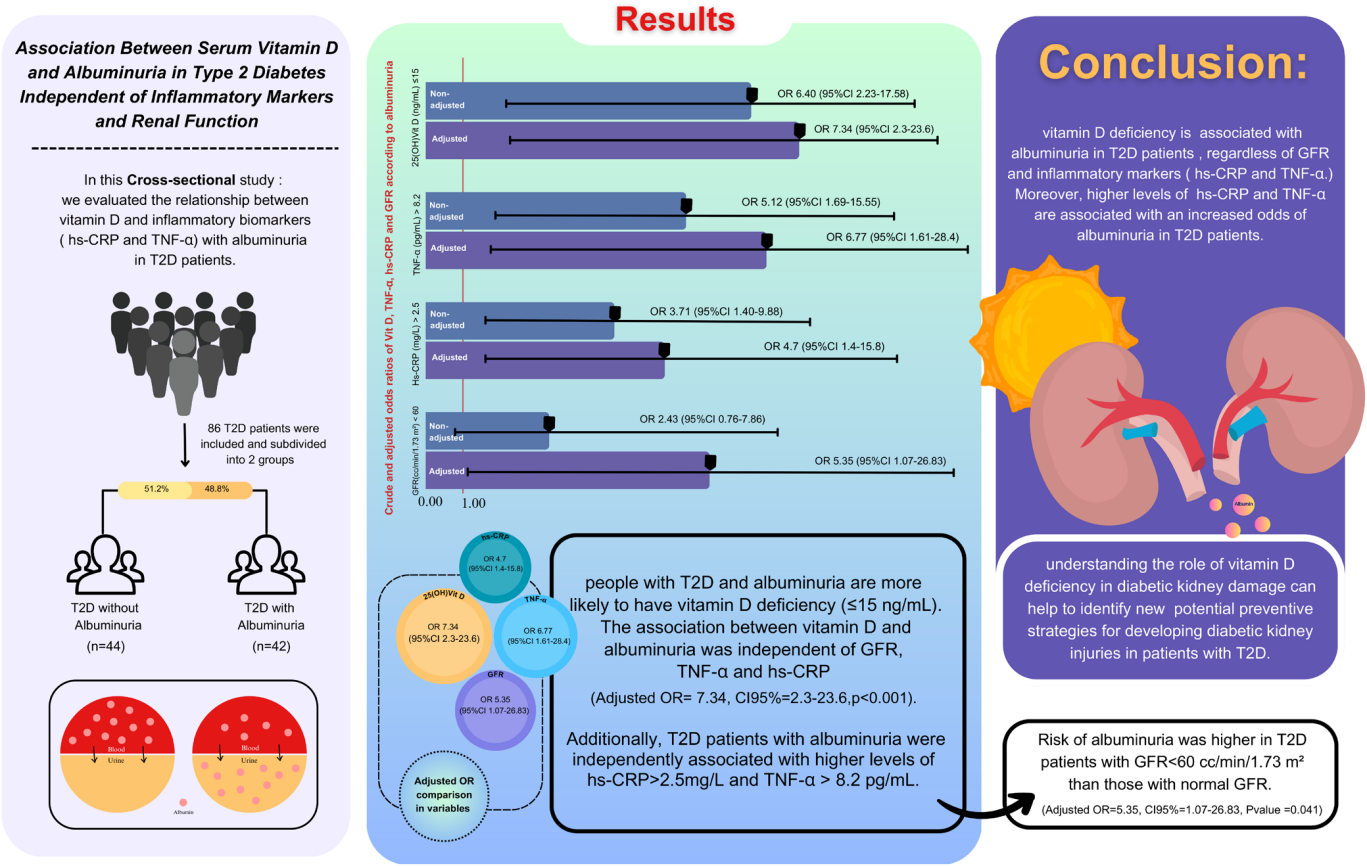
Graphical abstract illustrating the independent association between vitamin D deficiency and albuminuria in T2D, adjusted for renal function and systemic inflammation.

An overall summary of results and findings in this manuscript is concisely illustrated in Figure 2.

## 4 Discussion

In this study, we observed that T2D patients with vitamin D deficiency (25‐hydroxy vitamin D ≤ 15 ng/mL) were significantly more likely to have albuminuria, with an adjusted odds ratio of 7.34. Previous studies have also reported associations between low serum vitamin D concentrations and diabetic nephropathy in different populations, with odds ratios of 1.85 and 1.62 [27, 28]; however, the present research demonstrated a more robust association between vitamin D deficiency and albuminuria. While this finding supports the potential role of vitamin D as a biomarker for early renal involvement in T2D, the literature remains inconclusive. For instance, a recent study using Mendelian randomisation found no evidence supporting a causal relationship between vitamin D levels and diabetic nephropathy or kidney function in diabetic individuals [29]. Another study reported that although serum vitamin D levels tend to decline as nephropathy progresses, this association diminishes in advanced stages of the disease, particularly beyond the macroalbuminuria stage, where vitamin D no longer serves as a discriminating factor [30]. These discrepancies may reflect differences in study design, population characteristics, or disease staging, underscoring the need for further research to clarify the role of vitamin D in the pathogenesis and progression of diabetic nephropathy.

Furthermore, an important finding in this study, compared to other up‐to‐date research, is that vitamin D deficiency was strongly associated with the presence of albuminuria in T2D patients, independent of basic inflammatory markers (hs‐CRP and TNF‐α) and the GFR. Patients with chronic kidney diseases and a low GFR may experience vitamin D deficiency due to various mechanisms, including abnormal vitamin D metabolism resulting from disruption of renal function [31]. However, it is not explicitly comprehensible whether vitamin D deficiency in T2D patients with albuminuria is always accompanied by decreased renal function or whether albuminuria may occur in vitamin D‐deficient patients even in those with normal renal function. In this study, the association between vitamin D deficiency and albuminuria remained significant after adjusting for the eGFR. Additionally, the role of inflammation as a potential mechanism in the development of diabetic nephropathy has been proposed in recent studies [19, 20, 21]. Moreover, vitamin D is well known for its modulatory effect on inflammatory responses in the body [2, 3, 32]. One of the proposed mechanisms for developing diabetic nephropathy in vitamin D‐deficient individuals is inflammatory processes [32, 33]. For example, studies on rats with diabetic nephropathy demonstrated the local renal anti‐inflammatory effect of vitamin D receptor activation [34]. VDR agonists can also effectively ameliorate the progression of diabetic nephropathy by reducing renal inflammation [35]. Thus, it is also of prime importance to analyse the relationship between vitamin D and albuminuria, independent of basic inflammatory markers. The evidence from this study suggested that there is a strong association between vitamin D deficiency and albuminuria in T2D patients, irrespective of both the eGFR and inflammatory marker levels, such as the serum TNF‐α and hs‐CRP levels. Nevertheless, it should be noted that the assessment of inflammation in this study was limited to a few commonly used markers, and may not fully capture the complexity of the inflammatory processes involved in diabetic nephropathy. Further studies incorporating a broader panel of inflammatory biomarkers are warranted to comprehensively elucidate the underlying mechanisms linking vitamin D deficiency and albuminuria.

In addition to supporting the association of inflammatory biomarkers (hs‐CRP and TNF‐ α) with the presence of albuminuria in T2D patients, as also reported in previous studies [19, 20, 21], the evidence from our study identifies notable cutoff values that may help assess the risk of albuminuria. Our findings indicate that T2D patients with a serum hs‐CRP > 2.5 mg/L had 4.7‐fold higher odds of albuminuria compared to those with a hs‐CRP ≤ 2.5 mg/L. Similarly, T2D patients with a serum TNF‐α level > 8.2 pg/mL had 6.77‐fold higher odds of albuminuria than those with a lower serum TNF‐α level. These findings suggest that a serum hs‐CRP level greater than 2.5 mg/L or a serum TNF‐α level greater than 8.2 pg/mL may be independently associated with albuminuria in patients with T2D.

We observed a statistically significant but weak correlation between serum hs‐CRP and TNF‐α in patients with T2D without albuminuria (*r* = 0.34, *r* ^2^ = 0.1156; *p* = 0.023); while no association was observed between hs‐CRP and TNF‐α in patients with T2D with albuminuria. Moreover, a weak correlation was observed between serum hs‐CRP and BMI in patients with T2D with albuminuria (*r* = 0.3, *r* ^2^ = 0.09; *p* = 0.04), but even this weak correlation disappeared in patients with T2D without albuminuria. While these correlations reached statistical significance in some subgroups, they accounted for only a small proportion of the variance. Reporting *r* ^2^ values helps to clarify that, despite statistical significance, the relationships were biologically modest. These findings highlight the complexity of systemic inflammation in T2D, which is likely driven by the interplay of multiple genetic, metabolic, and environmental factors. Within such a multifactorial framework, low correlation coefficients are not unexpected and may reflect the influence of numerous unmeasured variables. Rather than being dismissed, these modest associations should be interpreted as indicative of the intricate and multifaceted nature of inflammatory processes in T2D [36, 37, 38].

To date, few studies have investigated the correlation between inflammatory markers and other variables in diabetic patients. They found a significant relationship between hs‐CRP and FBS, 2HPPG, HbA1C, TG, LDL‐C, and GFR in patients with T2D with and without albuminuria [39]. In our studied population, no remarkable association was detected between vitamin D deficiency and inflammatory markers such as serum hs‐CRP and TNF‐α in diabetic patients with or without albuminuria. In some but not all related studies, low levels of serum vitamin D have been associated with elevated inflammatory markers.

Currently, the main treatment for diabetic nephropathy is to control hyperglycemia and hypertension through lifestyle modifications and medications. Because current treatments do not thoroughly halt the development and worsening of renal damage in diabetic patients, the need to identify new therapeutic targets and supplemental policies for treating diabetic nephropathy is of prime importance. The significant association between vitamin D deficiency and albuminuria found in this study highlights the importance of further investigating the role of the protective effect of vitamin D in diabetic nephropathy. The current study illustrated that patients with vitamin D ≤ 15 ng/mL had 7.34‐fold higher odds of albuminuria than those with higher vitamin D levels. However, due to the cross‐sectional nature of the study, causal inferences cannot be made, and the potential protective effect of vitamin D requires confirmation through longitudinal or interventional studies. Notably, a recent meta‐analysis demonstrated a higher risk of nephropathy in vitamin D–deficient diabetic patients; however, when pooling data from interventional trials, vitamin D supplementation did not support a causal relationship with reduced nephropathy risk [40]. On the other hand, some literature suggests that the diagnosis and treatment of vitamin D deficiency may be associated with reducing insulin resistance and blood pressure, which are both adjustable risk factors for the development and progression of diabetic nephropathy [27]. These findings further emphasise the complexity of the relationship between vitamin D and diabetic nephropathy and highlight the necessity for well‐designed randomised controlled trials to clarify the clinical implications of vitamin D status in diabetic kidney disease.

Understanding the key features of inflammatory mechanisms involved in the development and progression of diabetic kidney injury will enable the identification of new potential targets and facilitate the design of innovative anti‐inflammatory therapeutic strategies. Future studies could include larger sample sizes and the inclusion of inflammatory biomarkers other than hs‐CRP and TNF‐α. By determining the exact role of inflammatory biomarkers in the pathogenesis of diabetic nephropathy, we could use these markers as indicators for the diagnosis of diabetic nephropathy in its initial stages and devise new strategies based on the anti‐inflammatory effects of drugs for preventing diabetic nephropathy.

This study has some limitations that must be acknowledged. The relatively modest sample size of the participants may have limited the statistical power of the multivariate analyses and affected the precision of the estimated associations, and the generalisability of the findings to broader patient populations. Although rigorous multivariate analyses and careful adjustment for potential confounders were employed to enhance the robustness of the results, the possibility of residual confounding cannot be entirely excluded. The cross‐sectional nature of the study inherently restricts the ability to infer causal relationships between vitamin D deficiency, albuminuria, inflammatory markers, and renal function. While the observed associations are strongly supported by established biological mechanisms and align closely with findings from previous longitudinal studies, we recognise that causality cannot be determined from a single time‐point assessment. Therefore, although causality cannot be established, these associations should be considered hypothesis‐generating and clinically relevant, paving the way for future prospective, longitudinal, and interventional studies in larger and more diverse populations to validate and expand upon these findings and better elucidate potential causal pathways. Despite these limitations, we believe that the present study adds meaningful evidence to the current understanding of the complex interplay between vitamin D status, inflammation, and renal dysfunction in T2D.

## Author Contributions

P.F. Conceptualization, Methodology, Investigation, Formal analysis, Writing – original draft, Writing – review & editing, Data curation. S.K. Conceptualization, Methodology, Investigation, Formal analysis, Writing – review & editing, Supervision, Data curation. M.A. Writing – review & editing. S.R. Methodology, Investigation, Formal analysis, Writing – review & editing, Supervision, Data curation. A.A. Investigation, Formal analysis, Writing – review & editing, Supervision. N.K. Writing – review & editing. S.K. Writing – review & editing, Project administration. E.S. Writing – review & editing, Visualization. M.N. Conceptualization, Methodology, Investigation, Formal analysis, Writing ‐ review & editing, Supervision.

## Ethics Statement

This research was approved by the ethics committee of Tehran University of Medical Sciences with the ethical code IR.TUMS.IKHC.REC.1398.180. This study was conducted in accordance with the Declaration of Helsinki.

## Consent

Written informed consent was obtained from all human subjects and/or their legal guardians.

## Conflicts of Interest

The authors declare no conflicts of interest.

## Data Availability

The data that support the findings of this study are available on request from the corresponding author. The data are not publicly available due to privacy or ethical restrictions.
